# Serum Concentrations of F**2**-Isoprostanes and 4-Hydroxynonenal in Hemodialysis Patients in Relation to Inflammation and Renal Anemia

**DOI:** 10.4137/bmi.s363

**Published:** 2008-05-27

**Authors:** Ingrid Wiswedel, Daniela Peter, Andreas Gardemann, Francesco Carluccio, Hannelore Hampl, Werner Siems

**Affiliations:** 1 Department of Pathological Biochemistry, Otto-von-Guericke-University of Magdeburg; 2 KortexMed Research Institute of Physiotherapy and Gerontology, Bad Harzburg; 3 Department of Nephrology and Medical Intensive Care, Charité University Clinic of Berlin, Germany and; 4 CNR-IFC, National Council of Research Institute of Clinical Physiology, Pisa, Section of Lecce; A.U.S.L/LE, Lecce, Italy

**Keywords:** F_2_-isoprostanes, end-stage renal disease (ESRD), hemodialysis patients, C-reactive protein, 4-hydroxynonenal, renal anemia

## Abstract

**Background:**

Patients with end-stage renal disease (ESRD) undergoing hemodialysis (HD) are apparently exposed to enhanced oxidative stress and to inflammation. It was the aim of this study to characterize the state of systemic oxidative stress of ESRD patients before and following HD using highly specific biomarkers, F_2_-isoprostanes and 4-hydroxynonenal (HNE). Furthermore the question should be answered, if there are associations between inflammation and systemic oxidative stress and/or between systemic oxidative stress and renal anemia, which is more or less typical for HD patients.

**Patients and methods:**

Concentrations of F_2_-isoprostanes, HNE, C-reactive protein (CRP) as marker of inflammation, and hemoglobin were measured in serum samples of patients with ESRD before and after HD and of healthy control persons for comparison. Total (esterified plus free) F_2_-isoprostanes were quantified by highly sensitive gas chromatography/mass spectrometry technique, HNE by thin layer chromatography and HPLC/UV detection, CRP by immunoturbidimetry and hemoglobin by clinico-chemical routine assay.

**Results:**

1. HD patients showed significantly higher serum concentrations of F_2_-isoprostanes and HNE than healthy human control subjects. 2. Total (esterified plus free) F_2_-isoprostane levels before HD were not significantly different from those after HD, whereas HNE levels were significantly decreased in patients after HD. 3. F_2_-isoprostane concentrations in HD patients correlated with the levels of CRP, whereas HNE concentrations inversely correlated with the content of hemoglobin.

**Conclusion:**

Both, F_2_-isoprostanes and HNE serum concentrations are useful oxidative stress parameters in ESRD patients undergoing HD. Whereas HNE strongly correlates with the severity of renal anemia, leading to left heart insufficiency, F_2_-isoprostanes (sum of free plus esterified) highly correlate with the degree of inflammation.

## Introduction

Hemodialysis (HD) in end-stage renal disease (ESRD) patients is associated with the development of oxidative stress, caused by the impairment of antioxidant defences and the bioincompatibility of the dialysis technique contributing to cardiotoxic effects (Canaud et al. 1999).

Reactive oxygen species (ROS) are known to play a role in the pathogenesis and progression of chronic renal failure (CRF). Apart from the kidney disease itself and the uremia, numerous other factors appear to contribute to oxidative stress (Trachtman et al. 1992), including HD via contact activation of neutrophils by the artificial surfaces of the dialysis membrane and the tubing system (Westhuyzen et al. 1995).

Increased levels of oxidants (e.g. malonic dialdehyde) and lower levels of antioxidants (glutathione, α-tocopherol) in CRF patients play an important role in the development of endothelial dysfunction, atherogenesis and cardiovascular disease (Loughrey et al. 1994; Delmas Beauvieux et al. 1995; Ceballos-Picot et al. 1996; Cavdar et al. 1997; Peuchant et al. 1997; Sommerburg et al. 1998; Ludat et al. 2000; Siems et al. 2002a; Siems et al. 2002b). A more specific biomarker of oxidative stress represents HNE, one of the major end products of lipid peroxidation generated mainly from arachidonic acid. HNE has cytotoxic and mutagenic properties, induces apoptosis, NF-κB activation and inhibition of enzyme activities (Esterbauer et al. 1991; Sommerburg et al. 1993; Grune et al. 1994; Siems et al. 1996; Sommerburg et al. 1998; Uchida et al. 1999; Ruef et al. 2001).

Previously, first reports on F_2_-isoprostanes as indicators of lipid peroxidation in renal diseases were published (Spittle et al. 2001; Handelman et al. 2001; Ikizler et al. 2002; Lim et al. 2002; Danielski et al. 2003; Ferraro et al. 2003; Kim et al. 2004; Wiswedel et al. 2005). F_2_-Isoprostanes have been established as chemically stable, highly specific and reliable biomarkers of *in vivo* oxidative stress, which can be very sensitively measured by gas chromatography/mass spectrometry (Morrow et al. 2002). Measurements in dialysis patients before dialysis showed higher plasma levels of F_2_-isoprostanes than in control subjects (Spittle et al. 2001; Handelman et al. 2001). But the data are not unique: in contrast to the data of Spittle et al. (2001) and of Handelman et al. (2001), Dogra et al. (2001) did not find an increase in plasma and urinary F_2_-isoprostanes in patients with nephrotic syndrome.

Both arachidonic acid oxidation products, F_2_-isoprostanes and HNE, are known to be involved in atherogenesis and myocardial injury (Blasig et al. 1995; Ruef et al. 2001; Morrow et al. 2002), which is the most critical point of life quality and mortality in CRF (Foley et al. 1995; Silverberg et al. 2000). There are also strong interactions between both types of cardiovascular risk factors, e.g. HNE induces the formation of 8-iso-PGF_2α_ in vascular smooth muscle cells (Ruef et al. 2001).

HD patients are not only exposed to enhanced oxidative stress, but also to inflammation (Spittle et al. 2001). Cardiovascular mortality remains high in ESRD patients, particularly in the group with evidence for inflammation (Yeun and Kaysen 2000). Inflammation may enhance oxidative stress through increased free radical generation via activation of NADPH oxidase and may directly modify proatherogenic factors.

The aim of this paper is 1. to determine F_2_-isoprostanes and HNE in the serum of ESRD patients before and following HD and for comparison in healthy control persons and 2. to examine if the markers of oxidative stress are correlated to the degree of inflammation and/or to the degree of renal anemia.

## Patients and Methods

### Patients

Patients and probands were required to provide written informed consent before the start of the evaluation. The place of dialysis was at the dialysis centre of Scorrano, Italy. Inclusion criteria of patients were as follows: presence of renal anemia (hemoglobin concentration<11 g/dl, time on dialysis >6 months, treatment in the center >3 months, normal values of GOT, GPT, LDH and γ-GT. Among the egligible patients the anamnesis about their life style required to exclude 12 persons because of smoking. Further criteria of exclusion were: patients taking antioxidants, patients with acute infection, with considerable iron overload, with an acute phase of rheumatological disorders and with neoplasiae. Furthermore, HD patients without well-functioning vascular excess and patients treated by vitamin E-bounded membranes were excluded. Only one patient was excluded at the end of the dialysis study because of wrong sampling. 14 patients came with hypertension. We aimed a strict control of hypertension with a target blood pressure ≤130/80 mm Hg (β-receptor blocker, ACE-I inhibitors, angiotensin II receptor blocker). For the investigated patients the “dry” weight was defined by impedenziometry and the “dry” weight was reached at the end of the HD session. 12 patients had intima media thickness and five of them had also documented one or more carotid plaques measured by B-mode ultrasonography. For 11 patients a left ventricular hypertrophy was documented, 16 patients were treated with erythropoietin. All patients were treated three times/week. High flux dialysis filters consisting of polysulphone/polyacrylonitrile were used. The evaluation was done at the midweek dialysis session. The participants of the control group were healthy persons from the dialysis centers in Scorrano and Berlin (nurses, doctors, technicians etc.). F_2_-isoprostane concentrations (8-iso-PGF_2α_ and 9α,11α-PGF_2α_) and HNE in correlation to CRP and hemoglobin content were analysed in 14 non-diabetic HD patients with chronic renal failure (6 male, 8 female, middle age 59 ± 14 years). F_2_-isoprostanes before and following hemodialysis were compared in 19 non-diabetic ESRD patients (5 additional patients: 2 male, 3 female, from the same age group). There were also control groups for analysis of F_2_-isoprostanes with 65 (30 male, 35 female, middle age 55 ± 15 years) and for analysis of HNE with 131 healthy probands (77 male and 54 female, middle age 60 ± 10 years).

### Sample extraction, hydrolysis and purification of F**2**-isoprostanes

The determination of the concentrations of F_2_-isoprostanes (sum of esterified and non-esterified isomers) was carried out as described previously (Wiswedel et al. 2004) with some modifications. To hydrolyze esterified lipids, the serum samples (1.0 mL) were treated with 320 μL of KOH (1 mol/L) at 40 °C for 30 min. Thereafter, the samples were neutralized by addition of 3 mL HCl (0.1 mol/L), and the pH was adjusted to 2 with HCl (1 mol/L). 9α,11α-PGF_2α_-d_4_ (1 ng in 20 μL ethanol) was added as internal standard. The samples were centrifuged at 5,000 × *g* for 15 min, and the supernatant was applied onto a C18-cartridge, pre-washed with 5 mL of methanol and 5 mL of water. The cartridge was then washed with 10 mL HCl (0.1 mol/L) and 10 mL of acetonitrile/water (15/85, v/v). Isoprostanes were eluted from the column with 5 mL of n-hexane/ethyl acetate/2-propanol (30/65/5, v/v/v). The prostanoid extract was applied then onto a NH_2_-cartridge, pre-washed with n-hexane (10 mL). The column was successively washed with 10 mL of n-hexane/ethyl acetate (30/70, v/v), acetonitrile/water (90/10, v/v) and acetonitrile. Finally, F_2_-isoprostanes were eluted with 5 mL of ethyl acetate/methanol/acetic acid (10/85/5, v/v/v).

### Derivatization and GC-MS/negative ion chemical ionization analysis

The extracts from the NH_2_-chromatography step were evaporated to dryness under a stream of argon at 45 °C. The residues were reconstituted with 40 μL of pentafluorobenzyl-bromide (10% in acetonitrile) and 20 μL of *N,N*-diisopropylethylamine (10% in acetonitrile) and incubated at 45 °C for 30 min. Thereafter, 50 μL of *bis*-(trimethylsilyl) trifluoroacetamide (BSTFA) and 5 μL of N,N-diisopropylethylamine were added to the dried sample. The samples were kept at 45 °C for 45 min, the solvents were removed, and the samples were reconstituted in 40 μL isooctane containing 0.1% BSTFA. F_2_-isoprostanes were separated by GC/MS using the following temperature program: initial temperature of 175 °C for 2 min; with a rate of 30 °C/min to a final temperature of 270 °C maintained for 20 min; total run time: 25.2 min. Quantitative analysis was performed with ammonia as reagent gas using selected ion monitoring (SIM) of the carboxylate anion [M-181]^−^ at *m/z* 569 and 573 for F_2_-isoprostanes and 9α,11α-PGF_2α_-d_4_, respectively. The *m/z* 569 ion current chromatogram showed the occurrence of two major peaks in serum samples, peak I co-eluting with authentic 8-iso-PGF_2α_ and peak II co-eluting with authentic 9α,11α-PGF_2α_. Peak I is expected to contain exclusively F_2_-isoprostanes, whereas peak II may contain both, mainly F_2_-isoprostanes, but additionally cyclooxygenase-derived PGF_2α_ (Wiswedel et al. 2004).

The response factors for the isoprostane isomers were 1.00 and 0.55 for 9α,11α- and 8-iso-PGF_2α_, respectively (means of three separate determinations).

F_2_-isoprostane analyses were performed in triplicate throughout for each plasma sample.

### Determination of CRP and hemoglobin

CRP was measured by immunoturbidimetry (Grützmeier and von Schenck, 1989), and hemoglobin with spectrophotometric methods within the clinical laboratory routine parameters. CRP and hemoglobin concentrations were 16.6 ± 3.8 mg/L serum resp. 9.75 ± 0.64 g/dl (mean ± SEM n = 14) for HD patients and 3.3 ± 0.1 resp. 15.1 ± 0.4 (mean ± SEM, n = 131; p < 0.05 resp. p < 0.01; patients versus controls) for healthy control persons.

### 4-Hydroxynonenal (HNE) analysis

The measurement of HNE includes the modification of this aldehyde with dinitrophenylhydrazine, the thin layer chromatographic analysis of the dinitrophenylhydrazones in three groups, and finally the isocratic analysis of the dinitrophenylhydrazine derivatives of the 4-hydroxyalkenales (Esterbauer et al. 1982; Grune et al. 1994; Sommerburg et al. 1993). During the HPLC-analysis a mixture of methanol and water (4:1, v:v) was used as an eluent. The detection-wavelength was set to 378 nm. The HNE-standard was produced from the diacetale which was stored in a chloroform solution at −20 °C.

### Statistical analyses

All data are presented as mean ± SEM. Comparisons between independent groups (patients versus controls) have been performed with the two-sample t test, comparisons between dependent groups (before and after dialysis) with the t test for paired data. Correlations between variables have been investigated with linear regression and Pearson’s correlation coefficient. All tests have been carried out two-sided and P values below 0.05 were considered to be significant.

## Results

**Figure 1:**
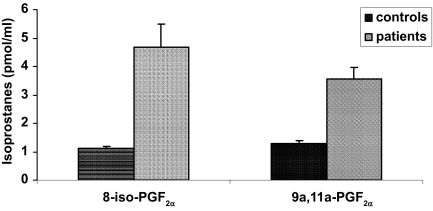
F_2_-Isoprostane concentrations (8-iso-PGF_2α_ and 9α,11α-PGF_2α_) in serum samples of patients (n = 19) and control persons (n = 65) Mean values ± SEM; two-sample t test; p < 0.001 versus control.

**Figure 2:**
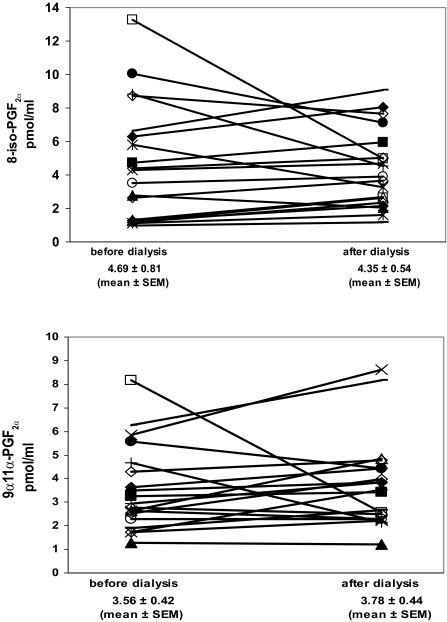
F_2_-Isoprostane concentrations [8-iso-PGF_2α_ (**a**) and 9α,11α-PGF_2α_ (**b**)] in serum samples of patients before and after hemodialysis (n = 19). The differences are not significant (t test for paired data).

Data concerning serum concentrations of F_2_-isoprostanes as biomarkers of the oxidative status of HD patients and normal control persons are shown in Figure 1. It can be seen that HD patients (4.69 ± 0.81 and 3.56 ± 0.42 pmol/mL) exhibited 4.2-fold (8-iso-PGF_2α_) and 2.8-fold (9α,11α-PGF_2α_) higher F_2_-isoprostane serum levels than healthy controls (1.12 ± 0.05 and 1.28 ± 0.11 pmol/mL). Furthermore, F_2_-isoprostane concentrations did not significantly change in patients before and following hemodialysis, as was shown in Figure 2a and 2b.

**Figure 3:**
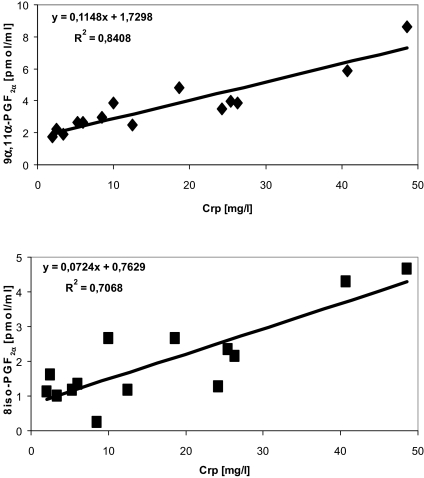
Correlation between levels of F_2_-isoprostanes [8-iso-PGF_2α_ (**a**) and 9a,11a-PGF_2α_ (**b**)] and C-reactive protein; n = 14 patients. Correlations have been investigated with linear regression analysis.

For 8-iso-PGF_2α_ and 9α,11α-PGF_2α_, correlations with a representative parameter of inflammation (CRP) and with a representative parameter of the degree of renal anemia (hemoglobin concentration) were examined. Figure 3 shows the correlations of F_2_-isoprostane concentrations [9α,11α-PGF_2α_ (Fig. 3a) and 8-iso-PGF_2α_ (Fig. 3b)] and CRP as parameter for inflammatory processes in the patients. We demonstrated a positive correlation of total (esterified plus free) F_2_-isoprostane isomers with the degree of inflammation, whereas no correlation between F_2_-isoprostanes and the degree of renal anemia (hemoglobin content) was found (not documented here).

In contrast, HNE, formed as one of the main degradation products of arachidonic acid, which is significantly increased in serum samples of ESRD patients (Fig. 4a), changed in dependence on hemoglobin concentrations, i.e. the HNE levels were higher with decreasing hemoglobin. It showed, therefore, an inverse relationship (significant negative correlation) with the degree of renal anemia (Fig. 4b). However, no significant correlation between the levels of HNE and the C-reactive protein (degree of inflammation) was observed (not shown).

## Discussion

### Chronic inflammation in HD patients

Many of ESRD and HD patients are in the state of chronic inflammation. The reason why renal failure is a source of inflammation has not been fully elucidated. A multifactorial origin of inflammation may be taken in consideration (Stenvinkel and Alvestrand, 2002). These include renal insufficiency (leading in the end stage to uremia) and its complications; as already established atherosclerosis, diabetes, hypertension; consequences of renal replacement treatment; back-leakage of endotoxins from contaminated dialysate and frequent infections (Tsirpanlis, 2005). Less biocompatible HD methods may contribute to the enhanced stimulation of neutrophiles and monocytes (through dialysis membrane interaction), the induction of cytokines, as IL-1, IL-6, TNFα and the increase of CRP (Morena et al. 2005). Even at initial stages of chronic renal failure, the CRP level is elevated (Tsirpanlis, 2005). CRP could be a sensitive risk index of the overall morbidity and mortality in CRF patients. As an acute phase protein with high sensitivity and low specificity, CRP is mainly synthesized in the liver and may be regulated largely by circulating levels of IL-6 (Thaunat et al. 2005; Vollenbroeker et al. 2005).

CRP has many features that may contribute to an inflammatory process: it binds to damaged cells and activates the complement system. It binds to atherogenic lipoproteins and induces aggregation of LDL and VLDL *in vitro*; and stimulates thrombus formation (Galle et al. 2003). Bergstrom et al. (2000) and Galle et al. (2003) reported that CRP is 5–10-fold higher in HD patients than in healthy controls (16.3 vs. 1.8 mg/L), which is associated with an increased cardiovascular risk. In the group of patients which we investigated the values were 16.6 vs. 3.3 mg/L, i.e. quite similar.

### Inflammation may be mainly responsible for oxidative stress

Inflammation is discussed as a well-documented factor influencing the development of oxidative stress in dialysis patients (Wratten et al. 2002). The way by which inflammation can contribute to oxidative stress in chronic renal failure is via activation of NADPH oxidase by interleukins and anaphylatoxins produced during HD sessions and/or myeoloperoxidase (Morena et al. 2005). Further sources of ROS are the mitochondrial respiratory chain, lipoxygenases, cyclooxygenases, xanthin oxidase, and NO synthase.

Both ROS and their secondary products are involved in the pathophysiological processes in ESRD. Oxidative stress, resulting from imbalance between oxidant production and antioxidative defence mechanisms, has been documented in ESRD patients using lipid, protein, nucleic acid and carbohydrate oxidative markers (Paleschi et al. 2007; Rutkowski et al. 2007; Coskun et al. 2007; Piroddi et al. 2007; Pai et al. 2007).

A highly significant positive correlation was observed between logCRP and reactive oxygen metabolites in HD patients by Samouilidou et al. 2007. F_2_-isoprostanes, associated with the inflammation process in patients on HD, positively correlated with CRP (Handelman et al. 2001; Ikizler et al. 2002) as also described in this paper. HNE, however, as readily diffusible molecule upon dialysis doesn’t follow the increase of CRP in chronic renal failure (Siems et al. 2002b and this study). Interestingly, an inverse correlation between HNE and hemoglobin serum concentrations could be demonstrated and therefore a role of HNE as predictor of hemolytic anemia was suggested.

### Esterified F**2**-isoprostanes may be not only reliable biomarkers of oxidative stress, but also mediators of inflammation

Measurement of F_2_-isoprostanes has advantages over other quantitative markers of oxidative stress: they are chemically stable, specific products of lipid peroxidation, they are formed *in vivo* and are present in all normal tissues and biological fluids (Montuschi et al. 2004). GC-MS is the reference analytical method for isoprostane measurement in biological fluids and tissues. F_2_-isoprostanes are widely accepted to correlate with the cardiovascular risk in human beings (Ikizler et al. 2002). This is an additional argument for the use of F_2_-isoprostanes in ESRD, which is closely connected with cardiac insufficiency.

F_2_-isoprostanes have been found to exert potent biological actions and therefore may participate as pathophysiological mediators of disease (Montuschi et al. 2004). In patients with lung diseases, 8-iso-PGF_2α_ concentrations in exhaled breath condensate reflect the degree of airway inflammation (Montuschi et al. 2000) with high levels reported in patients with chronic obstructive pulmonary disease and in smokers. 8-iso-PGF_2α_ is not only known as a pulmonary, but also as a potent renal vasoconstrictor. In the rat, F_2_-isoprostanes reduce glomerular filtration rate and renal blood flow by 40%–45% in the low nanomolar range (Takahashi et al. 1992). F_2_-isoprostanes have important *in vitro* activities that could be relevant to the pathophysiology of atherosclerosis. They promote platelet activation (Patrono and Fitzgerald, 1997) and induce mitogenesis in vascular smooth muscle cells (Fukunaga et al. 1993). Moreover, F_2_-isoprostane formation is increased during LDL oxidation *in vitro* and F_2_-isoprostanes are major contributors to the proadhesive effect induced by minimally oxidatively modified LDL on neutrophils (Fontana et al. 2002). F_2_-isoprostanes exert their biological effects by receptor-mediated interactions (acting as full or partial agonists at thromboxane receptors) and also by receptor-independent mechanisms (Montuschi et al. 2004).

F_2_-isoprostane serum levels of HD patients in this study were significantly increased versus healthy control persons. This is in agreement with other reports (Walter et al. 2000; Handelman et al. 2001; Lim et al. 2002). But, there were no significant differences of F_2_-isoprostane levels before and after HD, as was also found by Handelman et al. (2001), and in contrast to Kim et al. (2004), who observed 4-fold higher levels post hemodialysis.

Both human and experimental studies have indicated associations of F_2_-isoprostanes and severe inflammatory conditions, diabetes and atherosclerosis. Recently it was shown that intravenous administration of 8-iso-PGF_2α_ induced cyclooxygenase-mediated prostaglandin (PGF_2α_) formation and activation of inflammatory responses (Basu, 2004). In another recent study, 8-iso-PGF_2α_ induced IL-8 expression in human macrophages, a chemokine involved in inflammation and atherogenesis through mitogen-activated protein kinases (Scholz et al. 2003). These studies emphasize that F_2_-isoprostranes might be mediators of inflammation involving cyclooxygenases and/or cytokines and promote a possible link between oxidative stress and inflammation.

Increased concentrations of F_2_-isoprostanes are related with high concentrations of CRP. As in our measurements also in those of Spittle et al. (2001) and Handelman et al. (2001) was found that F_2_-isoprostanes correlate to the concentration of C-reactive protein as one of the most important factors in predicting cardiovascular mortality, which is by far the main cause of mortality in ESRD patients with cardio-renal anemia syndrome (Silverberg et al. 2003; 2004a, 2004b).

### Conclusions and outlook

In this study it was shown that ESRD patients undergoing hemodialysis are exposed to enhanced oxidative stress and to inflammation. The main limitations of this study were the relatively small number of patients and the obviously incomplete consideration of all parameters influencing inflammation and/or oxidative stress (e.g. lipid metabolism, blood pressure, cardiovascular disorders).

Biomarkers of oxidative stress, F_2_-isoprostanes and HNE, are significantly increased in the serum of patients and are highly correlated either to the degree of inflammation or renal anemia as well. Inflammation and oxidative stress may act synergistically to increase cardiovascular morbidity and mortality risk in the patients. The correction of anemia (e.g. by erythropoietin) reduces the oxidative stress to some extent, and also the cardiovascular risk. The prevention of oxidative stress in HD might, however, also focus on improving the hemocompatibility of the dialysis system, the supplementation of antioxidants and the modulation of NADPH oxidase by pharmacological approaches. In order to prove the effectiveness of therapeutic interventions, further efforts are obligatory. These include the enlargement and proper selection of suitable biomarkers that can be related to the pathogenesis and the development of the disease. Concerning F_2_-isoprostanes and partially HNE, most of the strict criteria for validation of the use of a biomarker *in vivo* (Biomarker Definition Working Group, 2001) are appropriate, but they only do reflect the ROS-mediated oxidation of arachidonic acid. Further limitations are 1. that the analysis of the used biomarkers (in particular F_2_-isoprostanes) is labor-intensive and requires expensive equipment and 2. an increase in F_2_-isoprostanes locally in tissues is not detected by measuring systemic oxidant stress. For HD patients it should be taken into account that small molecules, as HNE and possibly free F_2_-isoprostanes (but not esterified) are washed out during dialysis. It would be necessary, therefore, to follow up the serum or plasma concentrations of esterified F_2_-isoprostanes and those of other biomarkers, which have to be defined, very thoroughly in longitudinal studies.

## Figures and Tables

**Figure 4a:**
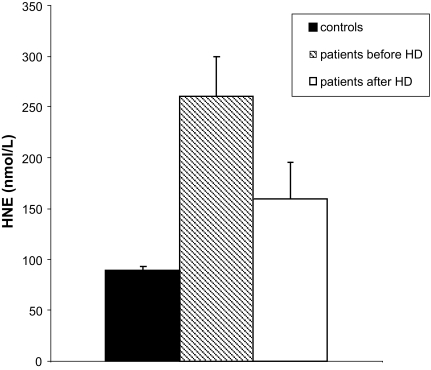
Serum concentrations of 4-hydroxynonenal in patients with chronic renal failure before and after hemodialysis (n = 14) compared to control persons (n = 131). Mean ± SEM, two-sample t test for comparison between patients and controls (p < 0.001; before HD vs. control and p = 0.002; after HD vs. control) and t test for paired data for comparison between patients before and after dialysis (p < 0.001).

**Figure 4b:**
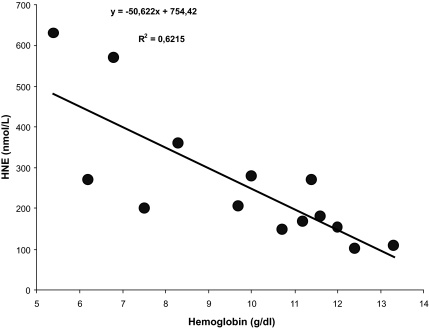
Correlation between HNE concentrations and hemoglobin content (n = 14 patients). Correlations have been investigated with linear regression analysis.

## References

[b1-bmi-03-419] Basu S 2004 Isoprostanes: Novel bioactive products of lipid peroxidation. Free Radic. Res 38 105 22 15104204 10.1080/10715760310001646895

[b2-bmi-03-419] Beauchamp MH Martinez-Bermudez AK Gobeil F 2001 Role of thromboxane in retinal microvascular degeneration in oxygen-induced retinopathy. J. Appl. Physiol 90 2279 99 11356793 10.1152/jappl.2001.90.6.2279

[b3-bmi-03-419] Bergstrom J Lindhol B Lacson E 2000 What are the causes and consequences of the chronic inflammatory state in chronic dialysis patients? Seminars Dialysis 13 163 75 10.1046/j.1525-139x.2000.00044.x 10833777

[b4-bmi-03-419] Blasig IE Grune T Schoenheit K 1995 4-Hydroxynonenal, a novel indicator of lipid peroxidation for reperfusion injury of the myocardium Am. J. Physiol., 269 (Heart Circ. Physiol) 38 H14 22 10.1152/ajpheart.1995.269.1.H14 7631842

[b5-bmi-03-419] Canaud B Cristol J Morena M 1999 Imbalance of oxidants and antioxidants in haemodialysis patients. Blood Purif 17 99 106 10449867 10.1159/000014381

[b6-bmi-03-419] Cavdar C Camsari T Semin I 1997 Lipid peroxidation and antioxidant activity in chronic hemodialysis patients with recombinant human erythropoietin. Scand J. Urol. Nephrol 31 371 75 9290168 10.3109/00365599709030622

[b7-bmi-03-419] Ceballos-Picot I Witko-Sarsat V Merad-Boudia M 1996 Glutathione antioxidant system as a marker of oxidative stress in chronic renal failure. Free Radic. Biol. Med 21 845 53 8902530 10.1016/0891-5849(96)00233-x

[b8-bmi-03-419] Coskun C Kural A Doventas Y 2007 Hemodialysis and protein oxidation products. Ann. N. Y. Acad. Sci 1100 404 8 17460205 10.1196/annals.1395.045

[b9-bmi-03-419] Danielski M Ikizler TA McMonagle E 2003 Linkage of hypoalbuminemia, inflammation, and oxidative stress in patients receiving maintenance hemodialysis therapy. Am. J. Kidney Dis 42 286 94 12900810 10.1016/s0272-6386(03)00653-x

[b10-bmi-03-419] Delmas Beauvieux MC Combe C Peuchant E 1995 Evaluation of red blood cell lipid peroxidation in hemodialyzed patients during erythropoietin therapy supplemented or not with iron Nephron 31 406 10 10.1159/000188510 7777104

[b11-bmi-03-419] Dogra G Ward N Croft KD 2001 Oxidant stress in nephrotic syndrome: comparison of F(2)-isoprostanes and plasma antioxidant potential. Nephrol. Dial. Transplant 16 1626 30 11477165 10.1093/ndt/16.8.1626

[b12-bmi-03-419] Esterbauer H Cheeseman KH Dianzani MU 1982 Separation and characterization of the aldehydic products of lipid peroxidation stimulated by ADP-Fe2+ in rat liver microsomes. J. Biochem 208 129 40 10.1042/bj2080129 PMC1153938 7159389

[b13-bmi-03-419] Esterbauer H Schaur RJ Zollner H 1991 Chemistry and biochemistry of 4-hydroxynonenal, malonaldehyde and related aldehydes. Free Radic. Biol. Med 11 81 128 1937131 10.1016/0891-5849(91)90192-6

[b14-bmi-03-419] Ferraro B Galli F Frei B 2003 Peroxynitrite-induced oxidation of plasma lipids is enhanced in stable hemodialysis patients. Kidney Int 63 2207 13 12753309 10.1046/j.1523-1755.2003.00008.x

[b15-bmi-03-419] Foley RN 1995 The prognostic importance of left ventricular geometry in uremic cardiomyopathy. J. Am. Soc. Nephrol 5 2024 31 7579050 10.1681/ASN.V5122024

[b16-bmi-03-419] Fontana L Giagulli C Cominacini L 2002 β_2_ Integrin-dependent neutrophil adhesion induced by minimally modified low-density lipoproteins is mainly mediated by F2-isoprostanes Circulation 106 2434 41 12417539 10.1161/01.cir.0000037223.92135.38

[b17-bmi-03-419] Fukunaga M Makita M Roberts LJ 1993 Evidence for the existence of F2-isoprostane receptors on rat vascular smooth muscle cells. Am. J. Physiol 264 1619 24 10.1152/ajpcell.1993.264.6.C1619 8333509

[b18-bmi-03-419] Galle J Seibold S Wanner C 2003 Inflammation in uremic patients: What is the link? Kidney Blood Press. Res 26 65 75 12771529 10.1159/000070986

[b19-bmi-03-419] Grützmeier S von Schenck H 1989 Four immunochemical methods for measuring C-reactive protein in plasma compared. Clin. Chem 35 461 63 2493344

[b20-bmi-03-419] Grune T Siems WG Zollner H Esterbauer H 1994 Metabolism of 4-hydroxynonenal, a cytotoxic lipid peroxidation product, in Ehrlich mouse ascites cells at different proliferation stages. Cancer Res 54 5231 35 7923145

[b21-bmi-03-419] Handelman GJ Walter MF Adhikarla R 2001 Elevated plasma F2-isoprostanes in patients on long-term hemodialysis. Kidney Int 59 1960 66 11318969 10.1046/j.1523-1755.2001.0590051960.x

[b22-bmi-03-419] Ikizler TA Morrow JD Roberts LJ 2002 Plasma F2-isoprostane levels are elevated in chronic hemodialysis patients. Clin. Nephrol 58 190 97 12356187 10.5414/cnp58190

[b23-bmi-03-419] Kalantar-Zadeh K Brennan ML Hazen SL 2006 Serum myelo-peroxidase and mortality in maintenance hemodialysis patients. Am. J. Kidney Dis 48 59 68 16797387 10.1053/j.ajkd.2006.03.047

[b24-bmi-03-419] Kim KM Jung BH Paen 2004 Alteration of F2-isoprostanes before and after hemodialysis in end-stage renal disease patients Prostaglandins, Leukot Essent Fatty Acids 70 475 78 15062851 10.1016/j.plefa.2003.10.001

[b25-bmi-03-419] Lim PS Chang YM Thien LM 2002 8-iso-Prostaglandin F_2_ as a useful clinical biomarker of oxidative stress in ESRD patients. Blood Purif 20 537 42 12566669 10.1159/000066962

[b26-bmi-03-419] Loughrey CM Young IS Lightbody JH 1994 Oxidative stress in hemodialysis. Q. J. Med 87 679 83 7820542

[b27-bmi-03-419] Ludat K Sommerburg O Grune T 2000 Oxidation parameters in complete correction of renal anemia. Clin. Nephrol 53 1 S30 5 10746803

[b28-bmi-03-419] Montuschi P Collins JV Ciabattoni G 2000 Exhaled 8-isoprostane as an in vivo biomarker of lung oxidative stress in patients with COPD and healthy smokers. Am. J. Respir. Crit. Care Med 162 1175 77 10988150 10.1164/ajrccm.162.3.2001063

[b29-bmi-03-419] Montuschi P Barnes PJ Roberts LJ 2004 Isoprostanes: markers and mediators of oxidative stress. FASEB J 18 1791 800 15576482 10.1096/fj.04-2330rev

[b30-bmi-03-419] Morena M Delbosc S Dupuy AM 2005 Overproduction of reactive oxygen species in end-stage renal disease patients: a potential component of hemodialysis-associated inflammation. Hemodial Int 9 37 46 16191052 10.1111/j.1492-7535.2005.01116.x

[b31-bmi-03-419] Morrow JD Zackert WE van der Ende DS 2002 Qantification of isoprostanes as indicators of oxidant stress in vivo Handbook of Antioxidants2nd edition Cadenas E Packer L Marcel Dekker, Inc New York, Basel 57 74

[b32-bmi-03-419] Pai AB Boyd AV McQuade CR 2007 Comparison of oxidative stress markers after intravenous administration of iron dextran, sodium ferric gluconate, and iron sucrose in patients undergoing hemodialysis Pharmacotherapy 27 343 50 17316146 10.1592/phco.27.3.343

[b33-bmi-03-419] Palleschi S De Angelis S Diana L 2007 Reliability of oxidative stress biomarkers in hemodialysis patients: a comparative study. Clin. Chem. Lab. Med 45 1211 18 17635073 10.1515/CCLM.2007.266

[b34-bmi-03-419] Patrono C FitzGerald GA 1997 Isoprostanes: potential markers of oxidant stress in atherothrombotic disease. Arterioscler. Thromb. Vasc. Biol 17 2309 15 9409197 10.1161/01.atv.17.11.2309

[b35-bmi-03-419] Peuchant E Delmas-Beauvieux MC Dubourg L 1997 Antioxidant effects of a supplemented very low protein diet in chronic renal failure. Free Radic. Biol. Med 22 313 20 8958156 10.1016/s0891-5849(96)00282-1

[b36-bmi-03-419] Piroddi M Depunzio I Calabrese V 2007 Oxidatively-modified and glycated proteins as candidate pro-inflammatory toxins in uremia and dialysis patients Amino Acids 32 573 92 17356806 10.1007/s00726-006-0433-8

[b37-bmi-03-419] Ruef J Moser M Bode C 2001 4-Hydroxynonenal induces apoptosis, NF-KB-activation and formation of 8-isoprostane in vascular smooth muscle cells. Basic Res. Cardiol 96 143 50 11327332 10.1007/s003950170064

[b38-bmi-03-419] Rutkowski P Slominska EM Szolkiewicz M 2007 Relationship between uremic toxins and oxidative stress in patients with chronic renal failure. Scand J. Urol. Nephrol 41 243 8 17469035 10.1080/00365590601017170

[b39-bmi-03-419] Salomon RG Batyreva E Kaur K 2000 Isolevuglandin-protein adducts in humans: products of free radical-induced lipid oxidation through the isoprostane pathway. Biochim. Biophys. Acta 1485 225 35 10832102 10.1016/s1388-1981(00)00038-x

[b40-bmi-03-419] Samoulidou E Grapsa E Kakavas I 2003 Oxidative stress markers and C-reactive protein in end-stage renal failure patients on dialysis. Int. Urol. Nephrol 35 393 97 15160547 10.1023/b:urol.0000022846.83505.3f

[b41-bmi-03-419] Samoulidou E Grapsa E Karpouza A 2007 Reactive oxygen metabolites: a link between oxidative stress and inflammation in patients on hemodialysis. Blood Purif 25 175 78 17215574 10.1159/000098521

[b42-bmi-03-419] Scholz H Yndestad A Damas JK 2003 8-isoprostane increases expression of interleukin-8 in human macrophages through activation of mitogen-activated protein kinases. Cardiovasc. Res 59 945 54 14553834 10.1016/s0008-6363(03)00538-8

[b43-bmi-03-419] Siems W Hapner SJ van Kuijk FJGM 1996 4-Hydroxynonenal inhibits Na^+^-K^+^-ATPase. Free Radic. Biol. Med 20 215 23 8746442 10.1016/0891-5849(95)02041-1

[b44-bmi-03-419] Siems W Quast S Carluccio F 2002a Oxidative stress in chronic renal failure as a cardiovascular risk factor. Clin. Nephrol 58 1 12 19 12227720

[b45-bmi-03-419] Siems W Carluccio F Grune T 2002b Elevated serum concentration of cardiotoxic lipid peroxidation products in chronic renal failure in relation to severity of renal anemia. Clin. Nephrol 58 1 20 5 12227722

[b46-bmi-03-419] Siems W Quast S Carluccio F 2003 Oxidative stress in cardiorenal anemia syndrome: correlations and therapeutic possibilities. Clin. Nephrol 60 22 30 12940531

[b47-bmi-03-419] Siems W Carluccio F Radenkovic S Grune T Hampl H 2005 Oxidative stress in renal anemia of hemodialysis patients is mitigated by epoetin treatment. Kidney Blood Press. Res 28 295 301 16534224 10.1159/000090184

[b48-bmi-03-419] Silverberg DS Wexler D Blum M 2000 The use of subcutaneous erythropoietin and intravenous iron for the treatment of the anemia of severe, resistant congestive heart failure improves cardiac and renal function and functional cardiac class, and markedly reduces hospitalizations J Am Coll Cardiology (JACC) 35 1737 44 10.1016/s0735-1097(00)00613-6 10841219

[b49-bmi-03-419] Silverberg DS Wexler D Blum M 2003 The cardio renal anemia syndrome: correcting anemia in patients with resistant congestive heart failure can improve both cardiac and renal function and reduce hospitalizations. Clin. Nephrol 60 93 102 12940539

[b50-bmi-03-419] Silverberg DS Wexler D Iaina A 2004a The role of anemia in congestive heart failure and chronic kidney insufficiency: the cardiorenal anemia syndrome. Perspect. Biol. Med 47 575 89 10.1353/pbm.2004.0072 15467179

[b51-bmi-03-419] Silverberg DS Wexler D Blum M 2004b The interaction between heart failure, renal failure and anemia—the cardiorenal anemia syndrome. Blood Purif 22 277 84 10.1159/000078698 15166489

[b52-bmi-03-419] Sommerburg O Grune T Klee S 1993 Formation of 4-hydroxynonenal and further aldehydic mediators of inflammation during bromotrichloromethane treatment of rat liver cells Mediators Inflammation 2 27 31 10.1155/S0962935193000031 PMC2365379 18475499

[b53-bmi-03-419] Sommerburg O Grune T Hampl H 1998 Does long-term treatment of renal anaemia with recombinant erythropoietin influence oxidative stress in haemodialysed patients. Nephrol. Dial. Transplant 13 2583 87 9794564 10.1093/ndt/13.10.2583

[b54-bmi-03-419] Spittle MA Hoenich NA Handelman GJ 2001 Oxidative stress and inflammation in hemodialysis patients. Am. J. Kidney Dis 38 1408 13 11728983 10.1053/ajkd.2001.29280

[b55-bmi-03-419] Stenvinkel P Alvestrand A 2002 Inflammation in end-stage renal disease: sources, consequences, and therapy Seminars Dialysis 15 329 37 10.1046/j.1525-139x.2002.00083.x 12358637

[b56-bmi-03-419] Takahashi K Nammour TM Fukunaga M 1992 Glomerular actions of a free-radical generated novel prostaglandin, 8-epi-prostaglandin F_2α_ in the rat. Evidence for interaction with thromboxane A2 receptors. J. Clin. Invest 90 136 41 1386085 10.1172/JCI115826 PMC443072

[b57-bmi-03-419] Thaunat G Beaumont C Chatenoud L 2005 Anemia after late introduction of sirolimus may correlate with biochemical evidence of a chronic inflammatory state Transplantation 80 1212 19 16314788 10.1097/01.tp.0000179106.07382.6a

[b58-bmi-03-419] Trachtman H Wilson D Rao PS 1992 The role of oxygen-free radicals in the development of chronic renal failure. Life Sci 50 1877 83 1317940 10.1016/0024-3205(92)90548-4

[b59-bmi-03-419] Tsirpanlis G 2005 The pattern of inflammation and a potential new clinical meaning and usefulness of C-reactive protein in end-stage renal failure patients. Kidney Blood Press. Res 28 55 61 15550763 10.1159/000082165

[b60-bmi-03-419] Uchida K Shiraishi M Naito Y 1999 Activation of stress signaling pathway by the end product of lipid peroxidation. 4-hydroxynonenal is a potential inducer of intracellular peroxide production. J. Biol. Chem 274 2234 42 9890986 10.1074/jbc.274.4.2234

[b61-bmi-03-419] Vollenbroeker B Koch JH Fobker M Suwelack B Hohage H Muller U 2005 Determination of cyclosporine and its metabolites in blood via HPLC-MS and correlation to clinically important parameters. Transplant. Proc 37 1741 44 15919451 10.1016/j.transproceed.2005.03.149

[b62-bmi-03-419] Walter MF Blumberg JB Dolnikowski GG 2000 Streamlined F_2_-isoprostane analysis in plasma and urine with high-performance liquid chromatography and gas chromatography/mass spectroscopy. Anal. Biochem 280 73 9 10805523 10.1006/abio.1999.4476

[b63-bmi-03-419] Wanner C Metzger T 2002 C-reactive protein a marker for all-cause and cardiovascular mortality in haemodialysis patients. Nephrol. Dial. Transplant 17 suppl 8 29 32 10.1093/ndt/17.suppl_8.29 12147774

[b64-bmi-03-419] Westhuyzen J Adams CE Fleming SJ 1995 Evidence for oxidative stress during in vitro dialysis Nephron 70 49 54 7617117 10.1159/000188543

[b65-bmi-03-419] Wiswedel I Hirsch D Kropf S 2004 Flavanol-rich cocoa drink lowers plasma F_2_-isoprostane concentrations in humans. Free Radic. Biol. Med 37 411 21 15223075 10.1016/j.freeradbiomed.2004.05.013

[b66-bmi-03-419] Wiswedel I Hirsch D Carluccio F 2005 F_2_-Isoprostanes as biomarkers of lipid peroxidation in patients with chronic renal failure Biofactors 24 201 8 16403981 10.1002/biof.5520240124

[b67-bmi-03-419] Wratten ML Galaris D Tetta C 2002 Evolution of oxidative stress and inflammation during hemodialysis and their contribution to cardiovascular disease Antioxidants Redox Signaling 4 935 44 12573142 10.1089/152308602762197470

[b68-bmi-03-419] Yeun JY Kaysen GA 2000 C-reactive protein, oxidative stress, homocysteine, and troponine as inflammatory and metabolic predictors of atherosclerosis in ESRD. Curr. Opin. Nephrol. Hypertens 9 621 30 11128424 10.1097/00041552-200011000-00006

[b69-bmi-03-419] Biomarkers Definitions Working Group 2001 Biomarkers and surrogate endpoints: preferred definitions and conceptual framework. Clin. Pharmacol. Ther 69 89 95 11240971 10.1067/mcp.2001.113989

